# Brain stimulation enhances dispositional mindfulness in PTSD: an exploratory sham-controlled rTMS trial

**DOI:** 10.3389/fpsyt.2025.1494567

**Published:** 2025-04-29

**Authors:** Kaveh Rayani, Andrea Grabovac, Peter Chan, Stefanie Montgomery, Mohammad-Reza Ghovanloo, Matthew D. Sacchet

**Affiliations:** ^1^ Department of Psychiatry, Faculty of Medicine, University of British Columbia, Vancouver, BC, Canada; ^2^ Neurostimulation Program, Vancouver General Hospital, Vancouver, BC, Canada; ^3^ Department of Neurology, Center for Neuroscience and Regeneration Research, Yale University School of Medicine, New Haven, CT, United States; ^4^ Department of Psychiatry, Meditation Research Program, Massachusetts General Hospital, Harvard Medical School, Boston, MA, United States

**Keywords:** mindfulness, rTMS, PTSD, dorsolateral prefrontal cortex, FFMQ

## Abstract

**Objective:**

Post-traumatic stress disorder (PTSD) is characterized by hypervigilance, intrusive thoughts, negative mood, and avoidant behaviors. Therapies involving mindfulness have been shown to reduce PTSD symptoms and modulate brain function. Pharmacological and brain stimulation interventions are also effective for treating PTSD. Non-invasive repeated transcranial magnetic stimulation (rTMS) of the dorsolateral prefrontal cortex (DLPFC) has been shown to regulate mood and improve PTSD symptoms.

**Methods:**

This is a retrospective chart analysis of data collected pre-treatment, post-treatment, and at three-month follow-up in a single-site, double-blind, sham-controlled trial of right DLPFC rTMS. 31 participants diagnosed with PTSD were recruited for this pilot study. Over two weeks, 19 participants received ten sessions of either 1 Hz or 10 Hz stimulation, and nine received sham treatment.

**Results:**

Participants in the rTMS group had a significant reduction in total Five Facet Mindfulness Questionnaire (FFMQ) scores from baseline to post-treatment, this difference was no longer observed when a false discovery rate (FDR) correction was applied. However, a significant improvement was observed in the rTMS group from baseline to the three-month follow-up in total FFMQ score and nonreactivity. This change in mindfulness scores suggests a potential delay in onset of benefits.

**Conclusions:**

Based on our preliminary data, rTMS may improve levels of dispositional mindfulness and its specific subcomponents. Future studies could investigate brain stimulation to assess its utility for improving mindfulness and related health outcomes to reduce suffering related to PTSD. Moreover, application of this neurostimulation modality for improving mental illness and well-being more generally merits further exploration.

**Clinical trial registration:**

https://clinicaltrials.gov/study, identifier NCT01806168.

## 1 Introduction

Post-traumatic stress disorder (PTSD) is a debilitating psychiatric condition associated with significant healthcare costs (1). With over 7% lifetime incidence in the United States (2) and over 9% in Canada (3), this prevalent condition is associated with a significant disease burden. PTSD symptoms consist of re-experiencing traumatic events, behavioral avoidance, altered mood and cognition, and hypervigilance (4). The wide range of treatments for PTSD includes selective serotonin reuptake inhibitors (SSRIs) (5), Eye Movement Desensitization and Reprocessing (EMDR), Cognitive Processing Therapy (CPT), and Prolonged Exposure (PE) (6–8). These, and other, pharmacological and psychotherapy-based treatment options for PTSD are generally efficacious (9). However, recent metanalysis data suggests that on average, approximately 10% of patients have symptomatic relapse following behavioral therapy (10). Among veterans, remission is more elusive, with 78% continuing to receive treatment after 4 years (11).

Patients with PTSD may be more prone to relapse due to genetic and neurobiological factors that impact fear regulation and resilience to stress. Research by Girgenti et al. (2021) found a correlation between PTSD and reduced expression of GABA-related genes, which could modify synaptic function in the prefrontal cortex, altering emotional regulation (12). These molecular changes may contribute to weakened inhibitory control over the fear response, thus making it harder for patients to maintain treatment gains. Additionally, structural and functional alterations in the prefrontal cortex, amygdala, and hippocampus impair fear extinction, further increasing the risk of symptom recurrence. This study also highlighted sex differences, with women showing more widespread transcriptomic changes, which may explain their elevated risk of persistent PTSD symptoms and higher relapse rates (12).

To fill the gap, other treatment modalities such as neurostimulation are supported by an expanding body of literature and are now more readily accessible. Repetitive transcranial magnetic stimulation (rTMS) is a non-invasive brain stimulation technique that is well-tolerated and effective in treating several psychiatric conditions (13). rTMS utilizes energy from magnetic fields to generate waves of depolarizing neuronal current up to three centimeters below the skull’s surface. rTMS has been shown to increase cerebral blood flow, glucose metabolism, excitatory neurotransmitter release, and receptor availability (14). rTMS has been approved by the US Food and Drug Administration (FDA) and Health Canada to treat Major Depressive Disorder (MDD). MDD rTMS protocols have included low (1 Hz) frequency, high (10-20 Hz) frequency, or theta burst, and have targeted the right and/or left dorsolateral prefrontal cortexes (DLPFC) (15–17). Low-frequency stimulation protocols are generally thought to be inhibitory, while high-frequency protocols stimulate activity in targeted and connected cerebral regions (18, 19). Laterality of the stimulation is somewhat contentious with some studies not differentiating between the impact of left/right-sided stimulation (20). However, left-sided rTMS at the DLPFC is primarily used in the treatment of MDD (21) while right-sided stimulation is favored in anxiety disorders such as PTSD (22, 23). Other, seemingly discordant results between studies utilizing differing methodologies may be explained, at least in part by transcallosal inhibition; the principle whereby inhibitory rTMS over one cortex may disinhibit the other (24).

There is a high rate of MDD in those who suffer from PTSD. Given the symptom overlap between PTSD with MDD in such domains as: sleep, interest, guilt, concentration, and negative thinking or suicidal thoughts, we hypothesized that treating PTSD first would also have a significant impact on MDD symptoms. The NICE guidelines notably recommend that in comorbid PTSD and MDD, the PTSD be treated first (25). Therefore, we chose to use right-sided rTMS paradigms to treat PTSD in our study rather than the more established left-sided paradigms for treating MDD. A number of studies have established right-sided rTMS as being more commonly used and effective in treating PTSD, as summarized in a recent systematic review (26). In our study, the distribution of patients with MDD among the rTMS and Sham groups was not significantly different, thus the effect of this potential confounding factor is expected to be unidirectional among the groups. Moreover, our previous publication using the same patient cohort confirms no significant response in symptoms of depression measured through the QIDS-SR or HDRS-21 to either the 1 Hz or 10 Hz stimulation protocols compared to Sham treatment (27).

There is an expanding body of evidence in support of rTMS for the treatment of certain anxiety disorders including Generalized Anxiety Disorder (GAD), anxious depression, and Panic Disorder (PD) (28). PTSD shares similar neurobiological mechanisms to other anxiety disorders, making rTMS a promising alternative or adjunct in the treatment of patients suffering from any of these conditions (29).

rTMS application to the DLPFC may help regular neural circuits associated with and reduce PTSD symptom severity. Resultant enhancements in prefrontal-limbic interactions may improve emotional regulation (30). Moreover, positive psychological expectancies, such as: hope and optimism are also associated with lower PTSD symptom severity. Thus, a positive mindset may play a role in enhancing resilience and recovery from traumatic experiences. Given that treatments for PTSD tend to underutilize positive cognitions, treatments utilizing both positive thinking and neurocircuitry modifying effects of rTMS may enhance the benefits each tool offers on its own (31).

Mindfulness is described as the capacity to attend to the present moment with awareness while avoiding judgment of one’s inner experience (32). Trait mindfulness can be defined as an individual’s aptitude for dispositional mindfulness; the ability to pay and maintain attention to present experiences, while remaining open and non-judgmental, so that individuals can increasingly observe their negative emotions in a way that minimizes reactivity and experiential avoidance (33). Mindfulness-Based Interventions (MBIs) have been shown to positively impact mental and physical health (34), neurocognition (35), and social functioning (36). These interventions utilize scales such as the ubiquitous Five Facet Mindfulness Questionnaire (FFMQ) devised by Baer and colleagues to measure multi-dimensional components of mindfulness (37).

Mindfulness-based practices are associated with neuroplastic changes in brain centers including the DLPFC, which is a key node in post-traumatic affect regulation (38). Ochsner et al. utilized functional Magnetic Resonance Imaging (fMRI) to show correlation between activity in the DLPFC, altering affect regulation, and reappraisal of negative affective experiences (39). This publication suggests that reappraisal of negative affective experiences improves because of DLPFC intervention. Increased lateral prefrontal activation during reappraisal is associated with reduced amygdala activation and resultant decrease in negative affect. Interventions targeting the DLPFC are thus likely to enhance reappraisal strategies as this cortical region is known to play a critical role in cognitive restructuring (39).

Moreover, neuroimaging research has provided evidence that MBIs alter brain structure and function in areas including the DLPFC (40). We hypothesize that targeting these regions through direct neurostimulation could be a means to alter mindfulness in quantifiable ways.

The affect regulating centers of the limbic system are highly connected with the prefrontal cortex (41). Right-sided rTMS targeting the DLPFC decreases amygdala hyperactivity, lessening autonomic hyperactivity (22). Numerous randomized controlled trials (RCTs) utilizing rTMS of the DLPFC have shown a reduction in PTSD symptom severity (18, 19, 22, 23, 42, 43). Our previous results were in line with these findings (27). The current study utilizes data from the same patient cohort to explore the effects of DLPFC rTMS on mindfulness, which was quantified through the FFMQ sub-scores: observing, describing, acting with awareness, nonjudge, and nonreactivity. We sought to test the hypothesis that neurostimulation of the right DLPFC causes quantifiable improvements in dispositional mindfulness, correlating with the previously observed improvements in PTSD symptom severity (27). Total mindfulness, as well as nonjudge, acting with awareness, and nonreactivity are believed to correlate most strongly with PTSD symptomology (44). We tested the hypothesis that right DLPFC rTMS significantly improves these components of mindfulness.

## 2 Materials and methods

### 2.1 Trial design

Descriptions of the current study’s protocols have been previously published and are included in Leong et al. (27). Briefly, this study is a retrospective chart analysis of a subset of the data collected during a prior double-blind, randomized sham-controlled study (NCT01806168). This subset of data was not included in the previous study and the associated findings are presented here for the first time. In the original RCT, a random sequence generator was used to assign participants to the treatment and Sham groups, with twice as many assigned to the former. Individuals provided informed consent before participation. The study was approved by the Clinical Research Ethics Board of the University of British Columbia (H12-01578).

### 2.2 Participants

31 participants between the ages of 19 and 70 with a primary diagnosis of non-combat-related PTSD were recruited from the psychiatry outpatient and community programs of Vancouver Coastal Health between 2014 – 2018. Participants were initially recruited through psychiatrist referral and were assessed by telephone, followed by in-person screening with the Mini-International Neuropsychiatric Interview (MINI) to confirm the diagnosis (45). Some participants were recruited after attending an outpatient PTSD psychoeducation group before rTMS. Participants had not previously received formal group therapy or mindfulness-based therapy.

Participants were required to maintain the same psychotropic regimen for the four weeks preceding the trial through to the completion. Participants with a psychotic illness, bipolar I disorder, substance use disorder, except nicotine, in the three months preceding the study, borderline personality disorder, or antisocial personality disorder were excluded from the study. Participants with active suicidal ideation, unstable medical conditions, neurological disorders including previous stroke, seizure history, intracranial ferromagnetic objects, or implanted devices in the head/neck were also excluded from study participation.

The study took place at Vancouver General Hospital. The sample size of the study cohort is smaller than the initially recruited number of participants due to missing data points and attrition throughout the study. Among the 31 participants initially recruited, one dropped out after five treatments, one missed the last two treatments and the Three-month Follow-up, and one other participant missed the Three-month Follow-up. Among the remaining 28 participants, the primary prior trauma was reported as follows: 16 had sexual violence, 17 were exposed to actual or threatened death or serious injury, and two witnessed such incidents. Eight participants reported multiple traumatic events and five reported significant emotional trauma. Except for two participants, all concurrently met the criteria for MDD.

### 2.3 Procedure

A Magstim Super Rapid-2 (Magstim Company Ltd, United Kingdom) with a Double 70 mm Air Film Coil (Model 3910-00) rTMS system was applied by the same nurse for all participants. The nurse was not blinded to the treatment groups, while the patients and investigators were. Sham stimulation was delivered through a sham Magstim D70 Air Film Coil model 3950-00. The Sham system was identical in appearance to the active system and produced similar noise and vibratory stimulation. rTMS was first applied with minimal intensity over the motor cortex until visible contractions in the contralateral abductor pollicis brevis muscle were observed (46). The DLPFC was then assumed to be located 6 cm anterior along the parasagittal line from where the resting motor threshold (RMT) was located (21). Stimulation intensity was set to 120% of the RMT with half of the treatment group receiving 2,250 pulses at 1 Hz stimulation over 37.5 mins, and the other half of the treatment group receiving 3,000 pulses at 10 Hz over the same period. The two groups were combined to attain the sample size needed to detect biologically meaningful differences. Stimulation was delivered with a train duration of 4 seconds and an intertrain interval of 26 seconds. Treatments were performed each weekday over two weeks, for a total of ten sessions. No instructions were given to the participant regarding mental activities including mindfulness, during rTMS treatment, or at other times during the study. This protocol was consistent with previously published, comparable rTMS trials for PTSD (18).

### 2.4 Measures

Primary outcomes in the initial RCT focused on the rTMS-based modulation of PTSD symptoms at baseline “Pre-treatment”, after treatment (“Post-treatment”), and at Three-month Follow-up as assessed using the Clinician-Administered PTSD Scale-IV (CAPS-IV) (47). Secondary outcomes assessed at the same time points included the Hamilton Depression Rating Scale (HAM-D) (48). Self-reported measures included the PTSD Checklist for Civilians (PCL-C) (49), the Quick Inventory of Depressive Symptomatology (QIDS) (50), the Beck Anxiety Inventory (BAI), and the Generalized Anxiety Disorder Assessment (GAD-7) (51). The results of these assessments are included in the original publication by Leong et al. and will not be reported here (27). An additional secondary outcome measure and the focus of the current study, the FFMQ, was also completed by each participant during Pre-treatment, Post-treatment, and Three-month Follow-up (52).

All information necessary to evaluate the findings of the paper is included in the manuscript. Additional data can be provided by the corresponding author upon request.

### 2.5 Data analysis

All statistical analyses were completed using the JMP 15 software package (JMP Statistical Discovery LLC, Cary, NC). Descriptive statistics are included in **Table 1**. Wilcoxon’s signed-rank test was used to compare age and Pearson’s ChiSquare was used to compare gender, psychiatric comorbidities, and psychotropic medications between the treatment groups. JMP reports the test statistic *S* (which is the sum of the rank scores) for Wilcoxon’s and *X^2^* for Pearson’s Chi-square. These and the corresponding two-sided *P*-values are provided in **Table 1**.

**Table 1:** Demographic data.

Characteristic	rTMS Group	Sham Group	Test-statistic	*p*-value
Participants (*N)*	19	9		
Gender (F(%)/M(%); *X^2^*; *P*)	16(84.2%)/3(15.8%)	7(77.8%)/2(22.2%)	0.172	0.678
Age (Mean ± SD ***;*** *S*; *P*)	42.21 ± 12.39	49.56 ± 6.98	167.5	0.072
Psychiatric Comorbidities (*N (%); X^2^*; *P)*				
MDD	17 (89.5%)	9 (100%)	0.491	0.484
GAD	9 (47.4%)	3 (33.3%)	0.491	0.483
SP	7 (36.8%)	2 (22.2%)	0.598	0.439
PD	14 (73.7%)	6 (66.7%)	0.147	0.701
OCD	3 (15.8%)	0 (0%)	1.59	0.207
ED	1 (5.3%)	1 (11.1%)	0.315	0.575
ADHD	1 (5.3%)	0 (0%)	0.491	0.483
Medications (*N (%); X^2^*; *P)*				
SSRI	6 (31.6%)	3 (33.3%)	0.009	0.926
SNRI	5 (26.3%)	2 (22.2%)	0.055	0.815
Antipsychotic	5 (26.3%)	0 (0%)	2.88	0.090
Alpha-Blocker	3 (15.8%)	2 (22.2%)	0.172	0.678
Benzodiazepine	5 (26.3%)	3 (33.3%)	0.147	0.701

Demographic information and comparison of each variable between the rTMS and Sham groups are shown. The number of participants in each category is indicated along with the percentage they comprise within the rTMS or Sham group. For age, comparisons were made using Wilcoxon’s signed-rank test with a test statistic, *S* and corresponding *P*-value reported. For gender, psychiatric comorbidities, and medications, Pearson’s Chi-Square was used to compare Sham and rTMS groups, and the test statistic (*X^2^*) and *P*-value were reported. Abbreviations used are as follows: (MDD), Major Depressive Disorder; (GAD), Generalized Anxiety Disorder; (SP), Social Phobia; (PD), Panic Disorder; (OCD), Obsessive Compulsive Disorder; (ED), Eating Disorder; (ADHD), Attention Deficit Hyperactivity Disorder; (SSRI), Selective Serotonin Reuptake Inhibitor; (SNRI), Serotonin and Norepinephrine Reuptake Inhibitor.

**Figure 1:**
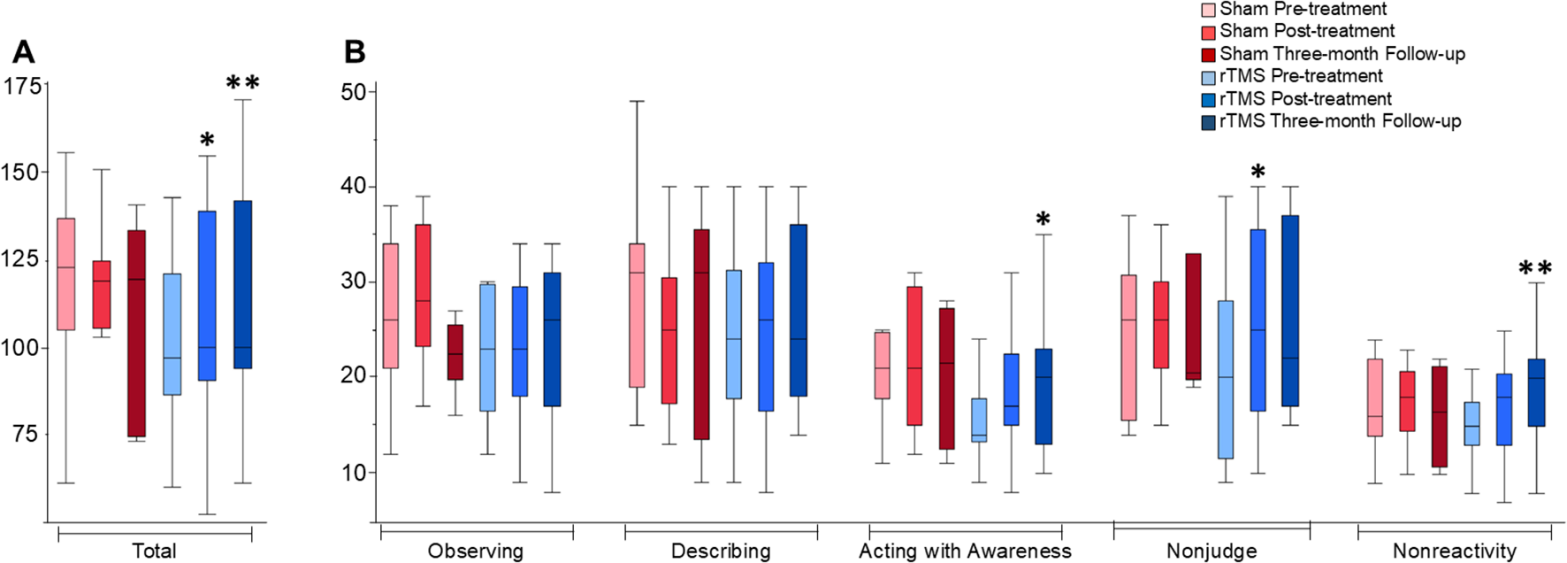
FFMQ total and sub-scores across time-points and groups. Box plots depict distributions of the FFMQ data. **(A)** includes FFMQ total scores, and **(B)** includes FFMQ sub-scores which are marked on the x-axis. * indicates a trend when comparing each data point to the baseline with statistical significance, *P* < 0.05. ** indicates the trend seen remained statistically significant after application of the FDR correction.

To provide an overview of the data distribution, the median and interquartile range (IQR) were calculated for each group at each time point and are reported in **Table 2** and plotted in **Figure 1**. This table also contains the data on the FFMQ total and sub-scores for each of the groups (Sham and rTMS) with an interaction term for each time point (Pre-treatment, Post-treatment, and Three-month Follow-up). One-sided tests were carried out as justified by the hypothesis (based on previous findings in the initial study by Leong et al., 2020) that the rTMS group would exhibit improvement in the FFMQ outcome measures compared to the Sham group (27). The *P-*values were corrected using a Benjamini-Hochberg false discovery rate (FDR) procedure across the total and five sub-scores. For each score, the FDR-corrected *P*-values are reported in the text below and in **Table 2**. Statistically significant results were set at *P <*0.05. FDR correction controls for the expected proportion of false discoveries, that is to say, it minimizes type I errors and ensures the most robust findings remain significant. If a p-value is no longer significant after the FDR correction, the initial uncorrected result may have been a false positive due to multiple testing.

**Table 2:** FFMQ results: pre-treatment, post-treatment, and three-month follow-up

		Sham			rTMS			Sham vs. rTMS			Post-treatment vs. Pre-treatment			Three-month Follow-up vs. Pre-treatment		
	Time Point	N	Median	IQR	N	Median	IQR	ChiSquare (*X^2^*)	*P*-value	FDR- corrected *P*-value	Test Statistic (*S)*	*P*-value	FDR- corrected *P*-value	Test Statistic (*S)*	*P*-value	FDR- corrected *P*-value
**Total**	Pre-treatment	7	123	32	16	97	34.75	2.16	0.142	0.450						
	Post-treatment	8	119	19.25	17	100	48.5	0.306	0.580	0.970	56.00	**0.034**	0.102			
	Three-month Follow-up	6	120	59	15	100	48	0.0243	0.876	1.000				55.50	**0.007**	**0.024**
**Observing**	Pre-treatment	7	26	13	16	23	13.25	0.702	0.402	0.460						
	Post-treatment	8	28	12.75	17	23	11.5	2.04	0.153	0.668	30.50	0.165	0.198			
	Three-month Follow-up	6	23	5.75	15	26	14	0.184	0.668	1.000				13.00	0.268	0.268
**Describing**	Pre-treatment	7	31	15	16	24	13.5	0.942	0.332	0.460						
	Post-treatment	8	25	13.25	17	26	15.5	0.0136	0.907	0.949	18.50	0.279	0.279			
	Three-month Follow-up	6	31	22	15	24	18	0.00	1.000	1.000				27.00	0.106	0.159
**Acting with Awareness**	Pre-treatment	8	21	7	16	14	4.5	6.38	**0.012**	0.058						
	Post-treatment	8	21	14.5	17	17	7.5	1.23	0.267	0.668	42.00	0.102	0.192			
	Three-month Follow-up	6	22	14.75	15	20	10	0.123	0.725	1.000				43.00	**0.040**	0.08
**Nonjudge**	Pre-treatment	8	26	15.25	16	20	16.5	1.22	0.270	0.460						
	Post-treatment	7	26	9	17	25	19	0.0040	0.949	0.949	60.50	**0.022**	0.102			
	Three-month Follow-up	6	21	13.25	15	22	20	0.0548	0.815	1.000				26.50	0.139	0.167
**Nonreactivity**	Pre-treatment	7	16	8	16	15	4.5	0.547	0.460	0.460						
	Post-treatment	8	18	6.25	17	18	7.5	0.219	0.640	0.949	35.50	0.128	0.192			
	Three-month Follow-up	6	17	10.5	15	20	7	0.501	0.479	1.000				51.50	**0.008**	**0.024**

The sample size (N), median, and interquartile range (IQR) for each group at each time point are included in the centermost columns of the table. At each time point (Pre-treatment, Post-treatment, and Three-month Follow-up) the total and five FFMQ sub-scores were measured. The “Sham vs. rTMS” column utilized the Kruskal-Wallis test to compare the treatment groups at each time point, reporting a ChiSquare and a corresponding *P*-value. At each time point, the Wilcoxon signed-rank test was used to obtain a test statistic, *S* and a one-sided *P*-values to test whether treatment-related improvement was greater in the rTMS group compared to the Sham group. False Discovery Rate (FDR) corrected *P*-values are included to account for multiple comparisons across the total and FFMQ sub-scores. Each row of the “Post-treatment vs. Pre-treatment” column includes group comparisons of FFMQ total and sub-scores between the Pre-treatment and Post-treatment. Each row of the “Three-month Follow-up vs. Pre-treatment” column similarly compares these time points using a pairwise test. Statistically significant results, *P* < 0.05 are bolded within the table.

The interaction between the time and treatment groups was the primary outcome. As such, for each treatment group, pairwise, repeated measures Wilcoxon signed-rank tests were used to separately compare each of the post-treatment scores and the Three-month Follow-up scores to Pre-treatment scores. This non-parametric test was used because of the relatively small sample size within each study group, negating the assumption of normality. Subsequent *post hoc* testing with the Kruskal-Wallis test was used to compare the FFMQ total and sub-scores for each time-point between the treatment groups and the test statistic, for which a ChiSquare (*X^2^*) is reported along with a *P*-value.

In a separate analysis, utilizing the methodological approach outlined before, we compared the 1 Hz and 10 Hz stimulation frequencies separately. The data is presented in the appendix to the text, **Table 1**.

## 3 Results

Demographics for the participants are presented in **Table 1**. There were no significant differences in gender, age, psychiatric comorbidities, or psychotropic medications between the treatment groups.

Pre-treatment, there were no significant differences in the total FFMQ scores between treatment groups (**Table 2**). The total FFMQ score between Pre-treatment and Post-treatment across treatment groups and the interaction between each variable and time was found to be significantly different. Findings are reported as median ± IQR, test statistic *S*, and *P*-value: (123 ± 32 Sham pre-treatment, 119 ± 19 Sham post-treatment vs. 97 ± 35 rTMS pre-treatment, 100 ± 49 rTMS post-treatment; *S* = 56.0, *P* = 0.034). However, after FDR correction the *P*-value was no longer statistically significant (*P*<0.05) (*P =* 0.034 pre-correction vs. *P* = 0.102 post-correction). The total FFMQ score between Pre-treatment and Three-month Follow-up across treatment groups was found to be significantly different while accounting for the interaction between the treatment and time variables (123 ± 32 Sham pre-treatment, 120 ± 59 Sham three-months post-treatment vs. 97 ± 35 rTMS pre-treatment, 100 ± 48 rTMS three-months post-treatment; *S* = 55.5, *P* = 0.007). After FDR correction, the *P*-value remained statistically significant (*P*<0.05) (*P =* 0.007 pre-correction vs. *P* = 0.024 post-correction), potentially indicating a time component in detectable improvements in the outcome.

Pre-treatment, there were no significant differences in the FFMQ sub-scores between treatment groups. A comparison of the FFMQ sub-scores between Pre-treatment and Post-treatment across treatment groups indicated a significant difference in the nonjudge sub-score (26 ± 15 Sham pre-treatment, 26 ± 9 Sham post-treatment vs. 20 ± 17 rTMS pre-treatment, 25 ± 19 rTMS post-treatment; *S* = 60.5, *P* = 0.022). Following the FDR correction, the *P*-value was no longer statistically significant (*P =* 0.022 pre-correction vs. *P* = 0.102 post-correction). The subsequent *post hoc* analyses were therefore exploratory in nature; a Kruskal-Wallis test was employed to explore potential differences within the subgroups. This analysis revealed a notable difference between the Pre-treatment and Three-month Follow-up scores. Specifically, when considering the interaction with time, significant differences were observed in the acting with awareness sub-score (21 ± 7 Sham pre-treatment, 22 ± 15 Sham three-months post-treatment vs. 14 ± 5 rTMS pre-treatment, 20 ± 10 rTMS three-months post-treatment; *S* = 43.0, *P* = 0.04) and the nonreactivity sub-score (16 ± 8 Sham pre-treatment, 17 ± 11 Sham three-months post-treatment vs. 15 ± 5 rTMS pre-treatment, 20 ± 7 rTMS three-months post-treatment; *S* = 51.5, *P* = 0.008). Following the FDR correction, for the acting with awareness sub-score, the *P*-value was no longer statistically significant (*P =* 0.04 pre-correction vs. *P* = 0.08 post correction). For the nonreactivity sub-score, the *P*-value remained statistically significant (*P =* 0.008 pre-correction vs. *P* = 0.024 post correction).

Given the potential for difference in response to various stimulation frequencies. The data was separated into subsets, accounting for 1 Hz vs 10 Hz stimulation. **Table 1** in the appendix presents this data. Between different stimulation frequencies, there were no significant differences either from baseline to post treatment, nor from baseline to three-months post-treatment. Trends exist, however, as in general the magnitude of response tended to be greater in the three-month follow-up time point, rTMS appears to have a greater response compared to Sham treatment, and 1 Hz appears to have a larger effect on total mindfulness and sub-scores, though given the small samples sizes in each group, none of these findings were statistically significant.

## 4 Discussion

The current study is preliminary in nature and expands on previous publications by exploring the utility of rTMS on trait mindfulness in a patient population with PTSD (42, 53). Our results indicate that rTMS targeting the right DLPFC increases levels of dispositional mindfulness, and mindfulness subcomponents both immediately following treatment and several months later. Immediately after treatment, we found a significant improvement in the nonjudge and total FFMQ scores following treatment, compared to the baseline between treatment groups. We also found a significant improvement in the acting with awareness and nonreactivity sub-scores and total FFMQ scores from baseline to the Three-month Follow-up between treatment groups. Yet, the difference in the acting with awareness sub-score was no longer seen following the FDR correction. However, differences were still detectable in the total FFMQ score and the nonreactivity sub-score following the FDR correction for multiple comparisons.

Acting with awareness is thought to be the central facet of mindfulness wherein changes in this component best predict improvement in mindfulness and mental health more generally (54). Among the mindfulness facets, nonjudge and acting with awareness have been shown to have the strongest negative relationship with negative affect (55). A recent meta-analysis showed that FFMQ total score, nonjudge, acting with awareness, and nonreactivity in this order have the highest correlation with PTSD symptomology (44). These are the sub-scores in which we most clearly observed a difference between the treatment groups.

These differences may be taken as trends, which, given the relatively small sample size, coupled with naturally large variability observed in biological data, did not reach the higher threshold for significance set by the FDR. The significant differences in the nonjudge and total FFMQ scores when comparing pre-treatment to post treatment were no longer seen after the correction, (26 ± 15 Sham pre-treatment, 26 ± 9 Sham post-treatment vs. 20 ± 17 rTMS pre-treatment, 25 ± 19 rTMS post-treatment; *S* = 60.5, *P* = 0.022 pre-correction vs. *P* = 0.102 post-correction), and (123 ± 32 Sham pre-treatment, 119 ± 19 Sham post-treatment vs. 97 ± 35 rTMS pre-treatment, 100 ± 49 rTMS post-treatment; *S* = 56.0, *P* = 0.034 pre-correction vs. *P* = 0.102 post-correction), respectively. However, given the nature of the FDR correction, this loss of significance does not necessarily mean the effect is absent, rather, it does not quite reach the adjusted, more strict statistical confidence threshold.

Comparing the three-month follow-up to pre-treatment, the acting with awareness sub-score was no longer seen after the FDR correction (21 ± 7 Sham pre-treatment, 22 ± 15 Sham three-months post-treatment vs. 14 ± 5 rTMS pre-treatment, 20 ± 10 rTMS three-months post-treatment; *S* = 43.0, *P* = 0.04 pre-correction vs. *P* = 0.08 post correction). Yet, the total FFMQ score (123 ± 32 Sham pre-treatment, 120 ± 59 Sham three-months post-treatment vs. 97 ± 35 rTMS pre-treatment, 100 ± 48 rTMS three-months post-treatment; *S* = 55.5, *P* = 0.007 pre-correction vs. *P* = 0.024 post-correction) and non-reactivity did remain significantly different after the FDR correction (16 ± 8 Sham pre-treatment, 17 ± 11 Sham three-months post-treatment vs. 15 ± 5 rTMS pre-treatment, 20 ± 7 rTMS three-months post-treatment; *S* = 51.5, *P* = 0.008 pre-correction vs. *P* = 0.024 post correction), potentially indicating a longitudinal component in the observable results as has been seen previously (19, 56).

Previous studies have shown that the observing sub-score may not correlate as strongly with the other four facets of mindfulness (44). Indeed, recent work by Mattes et al. suggests that observing is correlated with dissociation, absent-mindedness, and thought suppression, which are all PTSD symptoms (57). Considering these studies, it is not altogether unexpected that we were unable to identify a difference in the observing sub-score of mindfulness. Given that observing may be expected to correlate least closely with the total FFMQ and other sub-scores, we hypothesize that it is likely to be the least changed by MBIs or other therapies targeting trait mindfulness.

The current study is a continuation of our previous work where we found evidence that low-frequency right-lateralized rTMS alleviates PTSD symptoms (27). Here, we sought to investigate the effects of neurostimulation on mindfulness and its subcomponents. This treatment modality may be used as a substitute or adjunct for other interventions, namely MBIs which have long been known to support the management of PTSD symptoms (58). A recent meta-analysis indicates that greater levels of dispositional mindfulness are correlated with reduced PTSD symptoms (59). Lack of emotion regulation is the underlying cause of several psychopathological processes including anxiety and depression. The brain regions that contribute to affect regulation are therapeutic targets in several mood and anxiety disorders (54). As such, direct neurostimulation of these circuits may be a targeted means with the potential to yield a myriad of cognitive benefits (44).

Brain regions involved in mindfulness form complex and highly interconnected sets of networks (60). The medial prefrontal cortex (mPFC) is an important node in PTSD (41) and has been shown to interact with the hippocampus and amygdala, that are involved in affect processing (61–63). Neurobiological models of PTSD have proposed a reduction of top-down inhibition of these affect processing regions (64). Moreover, PTSD is thought to decrease cerebral metabolism in the mPFC and the anterior cingulate cortex, and conversely, to increase activity in the amygdala (61, 63). The mPFC also connects to the DLPFC making it a target of interest in PTSD (65, 66). Previous research suggests that the DLPFC in the right hemisphere may be more significantly impacted by PTSD (67).

Recent studies suggest that rTMS-induced changes in the DLPFC may modulate the default mode network (DMN) and salience network (SN). Given the central role of the DLPFC, rTMS stimulation here is thought to attenuate maladaptive connectivity patterns occurring in PTSD. Specifically, rTMS may decrease hyperconnectivity between the DMN and SN with resultant reduction in hypervigilance (68, 69).

A previous work by Liberzon and Abelson showed that right DLPFC stimulation through low-frequency rTMS modulates DMN connectivity, leading to decreased rumination and better attention control (69). In addition, rTMS associated changes in neurotransmitter release, receptor expression, and metabolic activity could play a role in underlying long-term effects on neural plasticity (14).

MBIs have been shown to downregulate DMN activity and enhance focus. The extent to which these interventions improve PTSD symptom improvement is unclear (70). It may be that mindfulness improvements occur independently of PTSD symptom reduction (71), or that these improvements act as a mediating mechanism for these changes (72). Enhanced mindfulness can facilitate cognitive flexibility and emotional regulation contributing to PTSD recovery independent of direct symptom reduction (73). The current study design does not allow for us to draw conclusions regarding interacting or parallel but distinct pathways of recovery. Thus, future studies or meta-analyses may seek to explore the existing body of literature.

This study has notable limitations. Alternatives to RMT such as neuronavigation can be used to more accurately target the DLPFC (74). Patients were asked to maintain psychotropic medications for 4 weeks before, through to the end of the rTMS course, however, treatment changes may have occurred during the Three-month Follow-up period in either group. Despite randomized study group allocation, at baseline, we found a difference in the FFMQ sub-score, acting with awareness between the treatment groups which was no longer seen after using the FDR correction for multiple comparisons. Future studies may use larger samples and/or pseudo-randomizing groups that account for baseline levels of mindfulness, avoiding issues which arise as a result of the exploratory nature of this pilot study. The relatively small sample size necessitated the pooling of data from patients who received low and high-frequency stimulation which may be a confounding variable. In support of this approach, a recent systematic review by Brown et al. (2025) found that there were no significant differences related to frequency when groups receiving active TMS were pooled in comparison to sham TMS (26). Given the exploratory nature of our study and small sample sizes, we have pooled the stimulation frequency subgroups to increase power in detecting differences between the treatment and sham groups. This work sought to explore the role of any stimulation on mindfulness parameters; future studies may further explore the role and mechanism of various stimulation frequencies in modulating PTSD symptom severity. While the current study is impressive in its application of rTMS within a randomized clinical trial design, the sample size limits confidence in the results; replicability should be assessed in future larger studies. Given the study design, we cannot elucidate causality of improvements in PTSD symptoms as these may be the primary factor affected by rTMS and secondarily result in improved mindfulness, or visa versa.

In summary, PTSD is a challenging disorder to treat and for some individuals, medications alone may not be sufficient to achieve symptom remission. Other treatment modalities such as neurostimulation, therapy, and MBIs present alternative options with potential additive benefits. In our sample of civilian participants, rTMS was found to have led to improvements in CAPS-IV and depressive symptoms (27). In this study, we tested the hypothesis that rTMS targeting the right DLPFC leads to improvements in FFMQ sub-scores reflecting changes in key mindfulness dimensions. Our results support this hypothesis and suggest that future research should continue to explore the impact of rTMS on outcomes related to mindfulness. It may be that concurrent MBIs before or after rTMS treatments may have a larger clinical impact and/or reduce symptomatic relapse. The protocol used here, which did not utilize MBIs, resulted in trends indicative of improvements in acting with awareness, nonreactivity, nonjudge, and total FFMQ scores. Even with rigorous correction for multiple comparisons through an FDR correction, significant differences were seen in the total FFMQ and nonreactivity sub-score at the three-month follow-up, suggesting a time-dependent response to rTMS. Overall, findings in this preliminary study suggests that rTMS may lead to improvements in key dimensions related to mindfulness, with clinical implications for overall PTSD symptomatology, and potentially for mindfulness in mood and anxiety disorders more broadly.

## Data Availability

The raw data supporting the conclusions of this article will be made available by the authors, without undue reservation.

## Appendix

**Appendix Table 1:** FFMQ results: pre-treatment, post-treatment, and three-month follow-up, separated by 1 Hz and 10 Hz stimulation.

	Treatment group	Frequency (Hz)	N	Pre-treatment		Post-treatment vs. Pre-treatment				Three-month Follow-up vs. Pre-treatment			
				Mean	Standard Error of the Mean	Mean Difference	Standard Error of the Mean	Test Statistic (*F)*	*P*-value	Mean Difference	Standard Error of the Mean	Test Statistic (*F)*	*P*-value
**Total**	Sham	1	4	101.50	14.01	5.75	8.66	1.16	0.35	17.67	10.35	2.53	0.10
	rTMS	1	8	101.25	21.66	12.50	6.12			21.43	6.77		
	Sham	10	3	139.00	16.09	-9.33	10.00			-15.00	12.67		
	rTMS	10	8	99.37	29.65	6.29	6.55			5.33	7.32		
**Observing**	Sham	1	4	24.75	9.43	2.50	2.15	1.18	0.34	-1.00	2.36	1.80	0.19
	rTMS	1	8	22.88	7.34	1.63	1.52			3.14	1.55		
	Sham	10	3	28.33	8.74	2.00	2.48			-0.50	2.89		
	rTMS	10	8	22.63	6.19	-1.71	1.62			-1.83	1.67		
**Describing**	Sham	1	4	23.50	7.72	-1.00	3.19	1.24	0.32	5.33	4.23	1.25	0.33
	rTMS	1	8	24.13	8.46	1.63	2.26			3.43	2.77		
	Sham	10	3	38.00	9.64	-6.67	3.69			-6.50	5.18		
	rTMS	10	8	29.13	19.50	-0.14	2.41			0.67	2.99		
**Acting with** **Awareness**	Sham	1	5	19.00	5.34	-0.40	2.03	1.32	0.30	-0.25	2.76	1.14	0.37
	rTMS	1	8	16.00	4.34	4.13	1.60			5.43	2.09		
	Sham	10	3	27.00	8.89	-0.33	2.61			3.00	3.91		
	rTMS	10	8	14.50	3.12	1.71	1.71			1.00	2.26		
**Nonjudge**	Sham	1	5	22.20	7.26	0.80	2.41	1.08	0.38	2.25	3.96	1.19	0.35
	rTMS	1	8	22.25	9.00	4.38	1.91			4.14	2.99		
	Sham	10	3	28.33	10.26	-1.00	3.81			-7.50	5.60		
	rTMS	10	8	18.87	8.98	4.86	2.04			3.50	3.23		
**Nonreactivity**	Sham	1	4	16.00	5.35	-0.25	1.89	0.28	0.83	2.33	2.45	2.35	0.12
	rTMS	1	8	16.00	2.62	0.75	1.34			5.29	1.61		
	Sham	10	3	17.33	5.77	2.00	2.19			-3.50	3.00		
	rTMS	10	8	14.25	4.40	1.57	1.43			2.00	1.73		

The sample size, mean, standard error of the mean (SEM) for each group at each time point are included in the leftmost columns of the table. With each of the stimulation frequencies analyzed separately, the Sham and rTMS groups were compared. A pairwise analysis was completed with test statistics (*F*) and *P*-values are presented in the columns, with each time point compared to the pre-treatment score separately. Each row of the “Post-treatment vs. Pre-treatment” column includes group comparisons of FFMQ total and sub-scores between the Pre-treatment and Post-treatment, mean difference and SEM are reported. Each row of the “Three-month Follow-up vs. Pre-treatment” column similarly compares these time points, with mean difference and SEM reported. False Discovery Rate (FDR) corrected *P*-values are included to account for multiple comparisons across the total and FFMQ sub-scores. Statistically significant results, *P* < 0.05 are bolded within the table.
